# An Artificial Intelligence Approach for Italian EVOO Origin Traceability through an Open Source IoT Spectrometer

**DOI:** 10.3390/foods9060834

**Published:** 2020-06-25

**Authors:** Simona Violino, Luciano Ortenzi, Francesca Antonucci, Federico Pallottino, Cinzia Benincasa, Simone Figorilli, Corrado Costa

**Affiliations:** 1 Consiglio per la Ricerca in Agricoltura e l’analisi dell’economia Agraria (CREA)—Centro di Ricerca Ingegneria e Trasformazioni Agroalimentari, Via della Pascolare 16, 00015 Monterotondo (Rome), Italy; simonaviolino@hotmail.com (S.V.); luciano.ortenzi@crea.gov.it (L.O.); francesca.antonucci@crea.gov.it (F.A.); simone.figorilli@crea.gov.it (S.F.); corrado.costa@crea.gov.it (C.C.); 2 Consiglio per la Ricerca in Agricoltura e l’analisi dell’economia Agraria (CREA)—Centro di Ricerca Olivicoltura, Frutticoltura e Agrumicoltura, Contrada Li Rocchi Vermicelli 83, 87036 Rende (CS), Italy; cinzia.benincasa@crea.gov.it

**Keywords:** VIS-NIR, ANN, made in Italy, minor components, pigments, antioxidants, non-destructive techniques, ready-to-use, spectral signature, artificial intelligence AI

## Abstract

Extra virgin olive oil (EVOO) represents a crucial ingredient of the Mediterranean diet. Being a first-choice product, consumers should be guaranteed its quality and geographical origin, justifying the high purchasing cost. For this reason, it is important to have new reliable tools able to classify products according to their geographical origin. The aim of this work was to demonstrate the efficiency of an open source visible and near infra-red (VIS-NIR) spectrophotometer, relying on a specific app, in assessing olive oil geographical origin. Thus, 67 Italian and 25 foreign EVOO samples were analyzed and their spectral data were processed through an artificial intelligence algorithm. The multivariate analysis of variance (MANOVA) results reported significant differences (*p* < 0.001) between the Italian and foreign EVOO VIS-NIR matrices. The artificial neural network (ANN) model with an external test showed a correct classification percentage equal to 94.6%. Both the MANOVA and ANN tested methods showed the most important spectral wavelengths ranges for origin determination to be 308–373 nm and 594–605 nm. These are related to the absorption of phenolic components, carotenoids, chlorophylls, and anthocyanins. The proposed tool allows the assessment of EVOO samples’ origin and thus could help to preserve the “Made in Italy” from fraud and sophistication related to its commerce.

## 1. Introduction

Extra virgin olive oil (EVOO) represents one of the most important ingredients of the Mediterranean diet, being used by most of the countries within the Mediterranean basin, owing to its excellent qualities and sensory properties ascribable to the fruits of olive trees (*Olea europaea* L.) [1]. The qualitative characteristics and the taste of EVOO are largely influenced by the olive plant varieties, the geographical origin, and the agronomic and production techniques employed as well. [2]. Recently, the consumption of EVOO has increased worldwide, even outside the Mediterranean and European countries (for example, India, Russia, China, and Australia). This trend demonstrates an increasing interest of both the producers and the consumers on the quality of food and calls for proper geographical identification and traceability of EVOOs [3].

The price of EVOO is on average 4–5 times higher than other vegetable oils. This is due to the higher production costs and to its higher nutritional and organoleptic properties. Therefore, the higher cost should in principle help to ensure high quality standards. On the other hand, the consumer is increasingly oriented towards the purchase of genuine food products with certified geographical origin [4]. In order to preserve the EVOO origin, the European Commission has established two types of certification relative to geographical origin and identification, namely the protected designation of origin (PDO) and the protected geographical indication (PGI) [4]. The definition of PGI refers to agricultural products and foodstuffs for which at least one stage of the production process must be carried out within a defined geographical area. For PDO, on the other hand, the entire production cycle must take place in a specific area. PGI labelling, therefore, focuses on quality and specific characteristics related to geographical origin [5]. As reported by the production regulations, in order to obtain the PDO certification, several conditions must be met such as a specific percentage of olive cultivars employed, well-defined cultivation practices, limited geographical areas of production, and specific characteristics regarding chemical and sensory properties of the final product. However, at the moment, to the best of our knowledge, there are no analytical parameters allowing a post hoc test on the actual geographical origin of PDO EVOOs. As a consequence, chemical and physical analyses are currently of limited use in the EVOO geographical certification [6].

Italy is one of the most important countries in the world in terms of olive oil supply and demand. Moreover, while the country boasts a large number of designations of origin labels, in total there are indeed forty-two PDOs of EVOO, the geographical indications, PGIs, exist for a limited number of three. The most active regional Italian realities are mainly located in the southern part of the peninsula, namely the region of Sicily with six PDOs and one PGI, Puglia with five PDOs, and Campania and Calabria with three PDOs each; however, there are also two important Italian central regions, such as Lazio and Tuscany, with four PDOs and one PGI, and five PDOs and one PGI, respectively [7].

Unfortunately, nowadays, one of the most common counterfeits to the detriment of the producer is the falsification of the EVOO geographical origin. Despite the great commitment and work of the authorities, counterfeiting is highly relevant for both the internal market and the global one, with difficulties in identifying and adopting convenient and reliable solutions. Indeed, due to lack of incentives, and often complex management to comply with, the technologies promoting product traceability are often difficult to implement.

Given the presence of consumers not aware of the fakes, an increasing number of low-quality olive oils often end up on the table, being not easily identifiable. The problems do not only concern consumers but also producers who operate correctly who are not economically damaged by the irregular practices of other companies [7]. Nevertheless, this type of counterfeiting causes enormous damages to the “Made in Italy” products’ image and to the economy of the country.

Fake EVOO bottles often report on the label incorrect information about the product or even refer to a totally different oil.

The “Made in Italy” products are represented by a set of values enabling the consumer to distinguish them from the foreign ones. However, sometimes their advertisement is used to lead the consumer to pay an even higher price for fake qualities relative to a forged product [8]. Thus, the consumer must also be aware of the differences between fake and authentic products, being guided through tools that allow them to distinguish “what is” from “what appears to be” [4].

However, within a globalized market, the fight against these counterfeits cannot be solely based on the enhancement of consumers’ awareness on the peculiarities and qualities that distinguish the “Made in Italy” from other products. Useful tools to contend with counterfeiting are those ensuring traceability [9], namely, the “possibility of reconstructing and following the path of a food in all phases of production, transformation and distribution” [10]. Therefore, traceability systems (technological and informative) are needed to strengthen and update a reliable information flow along the whole supply chain, simplifying consumers’ access to information. An infotracing traceability system can integrate information related to product quality with that regarding its traceability (physical and documentary), taking advantage of an online information system [9]. As reported by Violino et al. [11], EVOO traceability is not only important to define olive oil origin, but it is fundamental for the protection against fraud. In addition, innovative tools (e.g., radio frequency identification (RFID), near field communication (NFC), and QR code technologies in combination with blockchain systems) can be commercially implemented to verify the processes and could aid in controlling the quality of virgin olive oil. An example is represented by the use of the blockchain system (a distributed database of records in the form of encrypted blocks), where the developed online information (transactions) can be protected to proof against eventual alteration and fraud [12].

Nowadays, the traceability of food products has become a priority for both consumers, who are increasingly careful to buy healthy higher quality food, and producers. Indeed, traceability can guarantee the quality of raw materials, product certification, allow a rapid identification of problematic product lots, and permit the implementation of control systems to prevent fraud. Finally, food traceability is crucial to enhance transparency for a safer internationalization of the EVOO market, with consequent fair growth of the sector [13].

In the last decade, several analytical techniques have been developed to help the identification of olive oil [14,15], and about 200 compounds, out of hundreds, have been proved to be useful as compositional markers for traceability purposes of EVOO [16]. Compositional markers include both major and minor components. State of the art EVOO traceability approaches for geographical origin assessment are represented by major components determination (e.g., triacylglycerols, triglycerides, and fatty acids), stable isotopic ratio (e.g., ^13^C/^12^C in combination with ^18^O/^16^O), and multi-element characterization through the application of different multivariate statistical techniques [3]. Those commonly used for data analysis are cluster analysis, multidimensional scaling, artificial neural networks (ANNs), and partial least squares discriminant analysis (PLSDA).

The multi-element analysis carried out by Benincasa et al. [17] allowed for a correct classification of all the organic virgin olive oils under investigation collected from different Italian regions; however, as often visible in similar studies, the method showed a high, but not excellent, percentage of correct classifications. Another example is given by the stable isotope analysis made by Portarena et al. [18], reporting an *r* ranging from 0.76 to 0.80 in distinguishing the compositions of Italian monovarietal olive oils.

Numerous analytical techniques have focused on targeted approaches for the identification and quantification of pre-defined compounds, or classes of compounds. These include gas and liquid chromatography (GC and HPLC) coupled with mass spectrometry (MS) [19,20], nuclear magnetic resonance (NMR) spectroscopy [21], infrared spectroscopy [22], fluorescence [23], inductively coupled plasma mass spectrometry (ICP-MS) [17], and DNA-based methods [24]. Conversely, limited literature is available about the assessment of olive oil adulteration using non-targeted classification approaches, focusing on the detection of all compounds in a sample without a priori knowledge of chemical entities comparable with the reference of the pure sample fingerprint profile [25,26].

Recently, fundamental research has focused on the development of non-destructive techniques to reduce the use of solvents and reagents. This is done taking into account an international context of convergence towards higher environmental sustainability and an increased human health consciousness [27]. Among various non-destructive solutions aiming to fulfill these needs, near infrared spectroscopy (NIRS) has made major achievements. NIRS, paired with chemometric techniques, were satisfactorily used for olive oil authentication and screening [28,29,30,31,32,33]. Generally, using both software and hardware, open source infostructure solutions potentially result in significant cost reduction, making the scientific tools available for a wider audience [34]. Following this path, results such as the prediction of qualitative parameters, the evaluation of indices of different fruit and vegetable products [35], the authentication of olive oil according to the variety and geographical origin [36], and the detection of adulteration through acidic composition [37] were achieved using visible/near-infrared (VIS-NIR) spectroscopy.

The aim of this work was to assess the actual geographical origin of EVOOs labeled on the market as Italian and test the potential efficiency of an open source VIS-NIR device for traceability purposes. Indeed, the device could produce results for olive oil authentication (according to its variety and origin) and for the detection of fraud in a fraction of time and potentially on a much higher sampling number with respect to conventional analytical methods. In detail, the study pursued the goal of analyzing 92 Italian and foreign EVOO samples produced in 2018 and 2019. The samples were purchased from large commercial retailers and directly from olive mills (to ensure the true origin of the product). The spectral data were analyzed with an artificial intelligence model based on neural networks.

## 2. Materials and Methods

### 2.1. EVOO Samples

**Figure 1:**
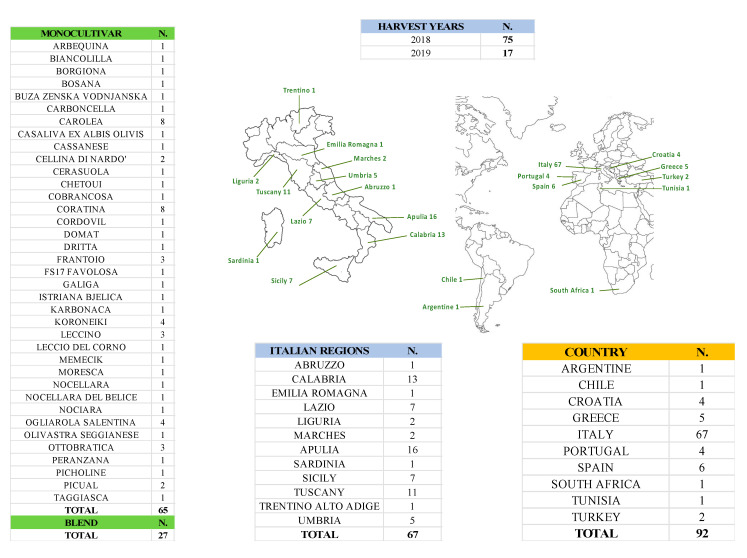
Monocultivar and blend extra virgin olive oil (EVOO) samples.

The study analyzed a total of 92 samples of Italian and foreign extra virgin olive oil (EVOO) owing to different cultivars, monovarietal (65) and blend (27), produced in two harvest years (2018 and 2019) (Figure 1).

The tested samples were bought from large retailers and directly from mills. Some samples were acquired specifically from the mills of the areas of Apulia, Calabria, and Sicily to ensure their origin. Other samples were sent, on a voluntary base, directly by the producers willing to participate in the research.

### 2.2. The Open Source IoT Spectrometer

**Figure 2:**
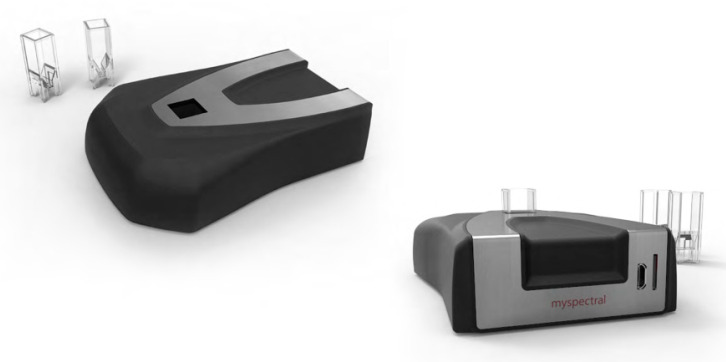
VIS-NIR ultra-compact spectrophotometer Lumini C Myspectral using standard cuvette holder for absorbance spectrophotometry.

The analyzed samples were stored and kept during the analyses at a controlled temperature of 16 °C. The samples, owing to the 2018 harvest campaign, were analyzed between March and May 2019 while those produced in the 2019 harvest campaign were analyzed between February and March 2020. The samples were scanned with a VIS-NIR spectrometer measuring and acquiring the spectral reflectance signatures for the EVOO samples for consequent qualitative evaluation. From each oil container (bottle or can) of the same sample, 12 spectral readings were acquired and afterwards averaged. The device used was the ultra-compact VIS-NIR spectrophotometer (Figure 2) Lumini C (Myspectral Ltd., Cambridge, MA, USA), able to measure spectral reflectance or absorbance. The device is small, light, low-cost, and open source. The spectral ranges covered 340–890 nm with an optical resolution equal to 8 nm and wavelength accuracy equal to 0.5 nm. The spectrophotometer is powered through a USB cable and stores data on connected cabled devices or on an internal micro SD card using a dedicated slot. For appropriate acquisition of the spectral signature, in relation to the sample reflectance characteristics, the acquisition can be set at different integration times. The system is equipped with its own internal illumination system.

**Figure 3:**
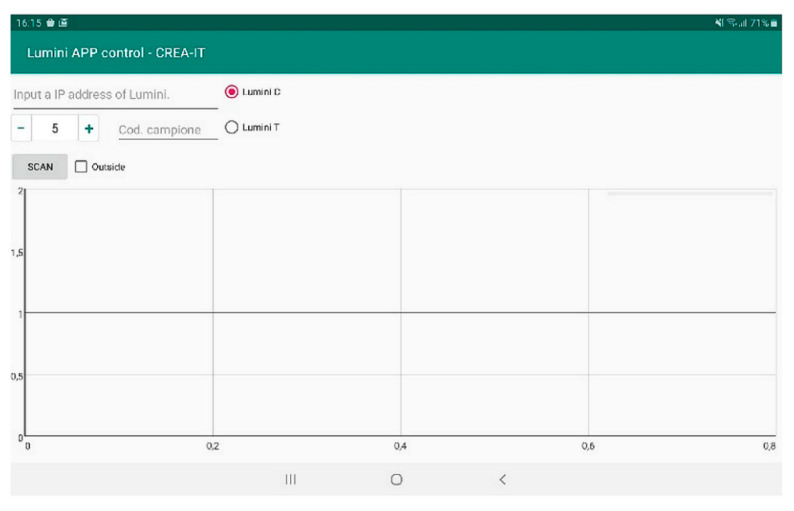
Screenshot of the Lumini app control CREA-IT for spectrophotometric acquisitions of EVOO samples.

A specific app was developed to manage and simplify the acquisition procedures. The software provided with the spectrophotometer, as commonly happens with open source technologies, was quite poor in terms of features and did not originally provide an appropriate historicization system for multiple acquisitions. For this reason, an app was developed and implemented. A screenshot of the app is reported in Figure 3.

**Figure 4:**
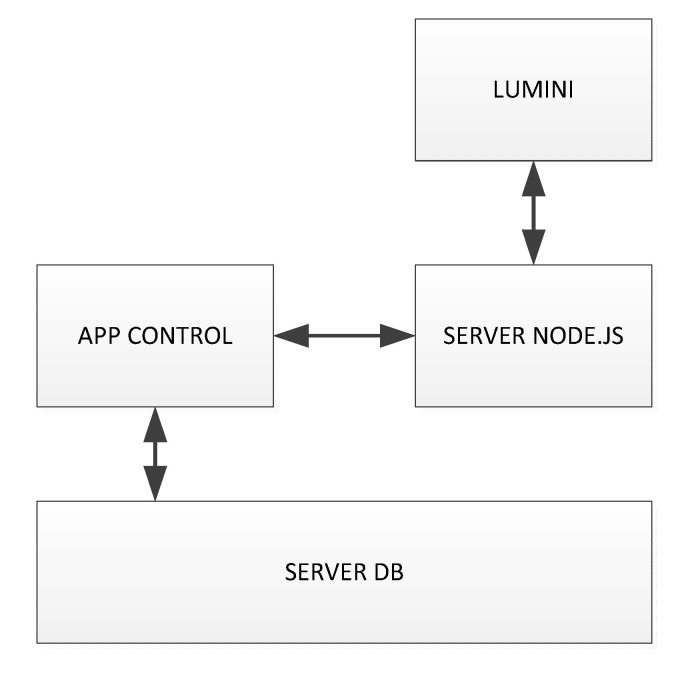
Block diagram of the Lumini C acquisition system via Android app.

The app was engineered considering two kinds of functions. The first (upper side of Figure 3) enables the configuration parameters of the instrument, such as the IP address, to connect the tablet to the device, the type of tool (in this case is Lumini C), the exposure time expressed in milliseconds (ms), and the sample’s name to be archived. The second (lower side of Figure 3), graphically represents the acquired spectrum for each scan. When a new sample name is entered, the graphic area is reset, ready to display the new spectra. This helps in case of incomplete or bad acquisition since it avoided losing samples’ values during the acquisition campaign. The app was developed using the Android environment and it is based on a client-server paradigm; on the client side there is the app, and on the server side there is the database for real-time storage of the spectrum and the node.js server to which the Lumini C is connected (Figure 4). The app software implements control mechanisms for the data stored on the database; these are essential since the data stored originally onboard within a microSD are now stored to a remote database. Through this mechanism, the data loss is minimized. In case of communication problems among the devices, the app notifies the problem and does not display the spectrum just acquired, allowing for a new scanning process.

### 2.3. Statistical Analysis

The multivariate matrix of Italian and foreign EVOO samples was analyzed with a 50–50 multivariate analysis of variance (MANOVA) procedure [38], a generalized multivariate Anova method based on principal component analysis (PCA) standardized data. The MANOVA was conducted in order to highlight significant differences between Italian and foreign VIS-NIR matrices. Adjusted *p*-values were conducted on a rotation testing based on 99,999 simulated datasets. The contribution of the variables was extracted for each rotation test [39].

An artificial intelligence approach was then applied in order to evaluate the possibility to classify Italian EVOOs and distinguish them from the foreign ones on the base of the 288 spectral transmittance values acquired through the VIS-NIR device. To do this, a multilayer feed forward artificial neural network (MLFN) was designed using a single hidden layer architecture with sigmoid hidden and SoftMax output neurons. The ANN was trained with the Bayesian regularization back propagation algorithm [40,41], as implemented in the deep learning MATLAB (The MathWorks, Inc., MA, USA) toolbox. The dataset was partitioned using 60 percent of the samples (55) as a training set and the rest as a test set (37). The test set was used to validate the model. This partitioning (equal for each soil group) was optimally chosen with the Euclidean distances calculated by the algorithm reported by Kennard and Stone [42], selecting parameters without a priori knowledge of a regression model. The cost function was minimized using the root mean squared (RMS) normalized error performance function with a 10^−8^ threshold on the gradient. In order to extract the most informative spectral transmittance values among the 288 acquired, in distinguishing Italian EVOO from foreign ones, it also conducted an analysis to study the feature importance. The hidden layer matrix (10 nodes × 288 variables) was a posteriori analyzed considering its elementwise absolute value. From the matrix was extracted the maximum value for each variable (e.g., column) obtaining a 1 × 288 row vector. The top 40 most significant spectral frequencies were chosen. The larger the value, the more relevant was the contribution to the ANN model. The model was developed using the MATLAB 9.7 R2019b Deep Learning Toolbox.

## 3. Results and Discussion

### 3.1. Artificial Intelligence Modeling Based on VIS-NIR Spectra

The MANOVA (50–50 MANOVA procedure) reported significant differences (*p* < 0.001) between the two Italian and foreign EVOO VIS-NIR matrices. The results of the analysis are reported in Table 1.

The ANN trained had a hidden layer size of 10 nodes and the algorithm converged after 976 iterations. Table 2 reports the characteristics and principal results of the ANN model used to predict Italian vs. foreign EVOO on the base of 288 VIS-NIR spectral transmittance data. All the 55 EVOOs in the training set were correctly classified. In testing, only five out of 37 samples were misclassified. These five samples consisted of two Italian commercial monocultivars (Coratina from Apulia and Taggiasca from Liguria) and three foreign blends from Greece, Argentina, and Croatia. Overall, 87 out of 92 samples (94.6%) were correctly classified.

The confusion matrix of the test set is reported in Table 3.

Overall, VIS-NIR spectroscopy analyses showed significant differences between Italian and foreign samples. From the results obtained through the ANN analysis, only five samples out of 37 were misclassified, e.g., two Italian commercial monocultivars (Coratina from Apulia and Taggiasca from Liguria) and three foreign blends (from Greece, Argentina, and Croatia). Probably, the two Italian samples were misclassified because of their uncertain geographical origin, considering that they are commercial oils. All the samples bought directly from the mills (noncommercial) were correctly classified. The off diagonal elements of the test confusion matrix (Table 3) are reported in Table 4.

Generally, machine learning relies on the amount of data for good modeling, where more data correspond to a modeling approach with increased robustness and performance. For this reason, even if the overall accuracy of the model is almost 90% and the convergence threshold of 10-8 on the RMS error gradient is very strict, the small size of the dataset (made of 92 samples) is not enough to validate the model. On the other hand, the high accuracy obtained despite the small dataset returns the reliability of the correlation observed [43].

The present work considered 67 Italian EVOOs and 25 foreign ones (two harvesting years: 2018 and 2019). However, it must be considered that other work using different methods to authenticate EVOO geographical origin were developed using a number of samples comparable and sometimes lower than that presented in this work. As reported by Bucci et al. [44], the data set for the statistical analysis was constructed on the results of the chemical analyses performed on 153 EVOOs (years of harvesting: 1997–1999), but finally only the samples produced in 1999 (53 oils) were analyzed in the laboratory. In the work conducted by Portarena et al. [45], they analyzed the isotopic composition and carotenoid content of 38 EVOOs from seven regions along the Italian coast using isotope ratio mass spectrometry (IRMS) and resonance Raman spectroscopy (RRS). The correlation between color and pigment content is well known in the literature [46]: the crushing of very green olives produces a typical green colored oil due to the high content in chlorophyll; if olives are more mature, carotenoids will prevail, determining a yellow-gold colored oil. Additionally, as the maturation progresses, the content and profile of phenolic compounds will also be affected: crushing green olives will result in an oil characterized by a higher content of phenolic acids, phenolic alcohols, oleuropein, and secoiridoids, whereas oils produced with dark brown olives will have a high content of anthocyanins, water-soluble plant pigments that take on different colors: red, blue, or violet [47,48].

### 3.2. Feature Importance

**Figure 5:**
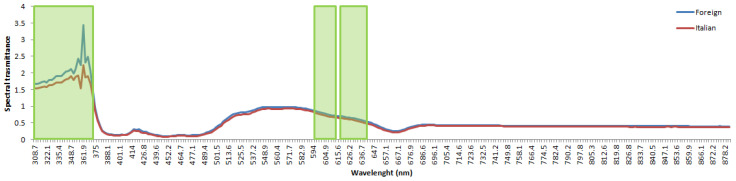
Mean VIS-NIR spectral data: Italian (red line), foreign (blue line). Higher importance spectral values extracted with the MANOVA rotation test are evidenced with green rectangles.

Observing the top 40 MANOVA rotation test’s most important variables (e.g., spectral lengths), the most informative ones ranged within the following frequencies: 308–373 nm, 594–612 nm, and 617–641 nm. The average VIS-NIR spectral data of foreign and Italian EVOOs are reported in Figure 5 together with the higher importance spectral values extracted with the aforementioned MANOVA rotation test.

**Figure 6:**
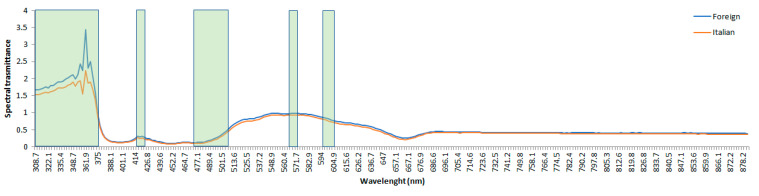
Mean VIS-NIR spectral data: Italian (red line), foreign (blue line). Higher importance spectral values in terms of artificial neural network (ANN) feature importance are evidenced with green rectangles.

Consequently, the 40 most important features extracted through the ANN procedure (e.g., spectral lengths), ranged within the following frequencies: 308–378 nm, 415–422 nm, 474–507 nm, 564–570 nm, and 596–605 nm. The average VIS-NIR spectral data of both Italian and foreign samples, together with the higher importance spectral values in terms of ANN feature importance, are reported in Figure 6.

The two feature importance approaches, MANOVA and ANN, evidenced common ranges of higher importance, which were: 308–373 nm and 594–605 nm. These spectral bands represent portions of the visible spectral range. The color of an oil is, therefore, due to the combination and proportion of its pigments [49]. These molecules do not depend only on the characteristics of the fruits (*Olea europaea* L.), the extraction processes used to produce the oil, and the conservation conditions [50] but, also, on weather and pedo-climate conditions [51]. Therefore, the relationship between the stage of ripeness and pigment content in EVOO could be, indeed, very important for further authentication studies [52].

The molecular structure of chlorophylls and, in particular, the planar structure of the tetrapyrrolic macrocycle coordinated by a magnesium ion, Mg^++^, is responsible for the absorption of visible light in the green region. Chlorophyll a gives a greenish-blue coloration, while chlorophyll b determines a yellowish-green color. The sensitivity of chlorophylls to extreme temperature and pH allows the formation of several distinct derivatives such as pheophytins, chlorophyllides, and pheophorbides. During the olive oil extraction process, the release of acids may cause pheophytinization reactions in the chlorophyll fraction, increasing the oils’ pheophytin content. The conversion of chlorophylls to Mg^2+^ free derivatives, such as pheophytins, where the Mg^++^ ion is replaced by two H^+^ ions, causes oil color changes over time [53,54,55]. Pheophytin a is present in greater quantities than pheophytin b. If olive oil is not well preserved, pheophytins can transform further, degrading to pyro pheophytin [55]. These latter can be considered an index of an aging oil. In addition to chlorophyll derivatives, pigments in extra virgin olive oil include carotenoids, the majority of which are lutein and carotene. Carotenoids are isoprenoid compounds with a hydrocarbon structure with various double bonds, C–C, which are responsible for their interesting properties as antioxidants [56]. Carotenoids can be further divided into carotenes (which contain only carbon and hydrogen atoms) and xanthophylls (which also contain oxygen atoms).

The spectra of olive oils analyzed in this work agree with those reported in the literature [57,58,59,60,61].

The peaks occurring in the range between 308 and 380 nm are mostly due to phenolic components [62]. In detail, we found the peak at around 350 nm, the absorption zone of flavones, present in the EVOO absorbance spectrum useful to distinguish Italian EVOOs from foreign ones. Flavonoids are plant secondary metabolites with different phenolic structures. These compounds are mostly used to generate pigments, which play an important role in the colors of plants producing yellow or red/blue pigmentation. Flavonoids such as apigenin, apigenin-7-*O-*glucoside, luteolin, luteolin-7-*O*-glucoside, luteolin-4-*O*-glucoside, diosmetin, quercetin, and quercetin-3-rutinoside are present in olive oils and contribute to the health benefits of consumers. The antioxidant and cellular damage repairing properties that make them useful for preventing cancer, cardiovascular disease, and degenerative diseases in general have been widely studied [63]. The main factors that contribute to their increase in the oil is the maturity index of the fruits and the degree of grinding and the malaxation conditions of the paste during the extraction processes of the oil [64].

The peaks occurring in the range between 415 and 422 nm are due to the compounds absorbing dark blue colored light, mainly carotenoids, as well as pheophytin a, pheophorbide a, and pyro pheophytin a [59], and are characterized by a yellow color.

The peaks occurring in the range between 474 and 507 nm are due to the compounds absorbing green/yellow colored light, and correspond to carotenoids, such as astaxanthin and canthaxanthin. In any case, the major carotenoids in olive oil are β-carotene and lutein, both of them providing several health benefits. Lutein exhibits antioxidant and anti-inflammatory activity protecting against DNA damage [65].

Moreover, the peaks occurring in the range between 564 and 570 nm and between 594 and 605 nm are due to the compounds absorbing orange colored light, characterized by purple/violet and green/blue colors, respectively, and corresponding to chlorophylls and anthocyanins.

## 4. Conclusions

Spectroscopic techniques paired to chemometric analyses are widely used to authenticate and differentiate edible oils. Most spectroscopic methods tend to focus on the major compounds of the saponifiable fraction of an oil, and only a few have been concentrating on the contents of minor compounds, such as pigments and antioxidants. The European community has not yet accepted many of the scientific community’s indications concerning minor compounds, which, by law, are not taken into consideration for the definition of EVOOs’ authenticity. However, many of the minor compounds are present in significant amounts only in EVOOs, and their quantification could greatly help the oil industry. Although further analysis will be needed to expand the case studies on olive oils, this work provides a clear indication of how pigment and antioxidant contents are crucial for the authentication and definition of the quality parameters of an EVOO. In detail, we found that the peak at about 360 nm and the broad band around 550 nm present in the EVOO absorbance spectrum can be used to distinguish Italian EVOO from foreign ones. As opposite to expensive and time-consuming chromatographic methods, procedures relying on (open source) spectroscopic instruments are cheap (less than 1000 €) and do not require sample preprocessing. Moreover, being fast, these techniques can be used to assess a huge collection of samples within a reasonable time. The quantitative analysis of pigments can take place directly at production sites and stores, through portable tools that are easy to use, even by non-expert staff. The trained ANN used to classify the samples according to their optical spectra can be easily implemented on an app for immediate classification. The development of simple and reliable methods that can verify the authenticity and guarantee the quality of agri-food products is crucial. Encouragingly, this type of analysis would be very beneficial for the producers themselves as well as consumers. Indeed, these techniques can score comparable precision with respect to the more expensive and time-consuming traditional ones. Moreover, since their application cost relies entirely on the instrumental budget, and not on reagent or other expensive consumable materials, they can be applied to a high number of samples and thus, in case of supposed fraud, can be used as pre-screening tools leading to time and economic optimization.

## Figures and Tables

**Table 1:** MANOVA results based on Italian and foreign EVOO samples.

Source	DF	exVarSS	nPC	nBu	exVarPC	exVarBU	*p*-Value
Italian vs. Foreign	1	0.04276	2	42	0.832	1	0.005056
Error	90	0.95724					

DF, degrees of freedom; exVarSS, explained variances based on sums of squares; nPC, number of principal components used for testing; nBu, number of principal components used as buffer components; exVarPC, variance explained by nPC components; exVarBU, variance explained by (nPC+nBU) components; *p*-value, the result from 50–50 MANOVA testing.

**Table 2:** Characteristics and principal results of the multilayer feed forward artificial neural network (MLFN) model (training and internal test) in predicting the classification of Italian vs. foreign EVOO: number of cases, training time, number of trials, and percentage of bad predictions.

**Training (60%)**	
Number of Cases	55
Number of hidden layers	1
Number of nodes	10
Training time	1:26:02
Number of trials	976
% bad predictions	0.0
**Testing (40%)**	
Number of cases	37
% bad predictions (N)	13.51 (5)

**Table 3:** Confusion matrix of the test set of the MLFN model used in predicting the classification of Italian vs. foreign EVOO. The correctly classified samples are reported on the main diagonal of the matrix.

	Italian	Foreign	Total
Italian	25	2	24
Foreign	3	7	8

**Table 4:** Off diagonal elements of the test confusion matrix reported in Table 3.

Origin	Cultivar	Commercial
Italy	Coratina	Yes
Italy	Taggiasca	Yes
Greece	Koroneiki	Yes
Argentina	Coratina	Yes
Croatia	Karbonaka	Yes
